# Combating Oxidative Stress and Inflammation in COVID-19 by Molecular Hydrogen Therapy: Mechanisms and Perspectives

**DOI:** 10.1155/2021/5513868

**Published:** 2021-10-04

**Authors:** Duried Alwazeer, Franky Fuh-Ching Liu, Xiao Yu Wu, Tyler W. LeBaron

**Affiliations:** ^1^Department of Nutrition and Dietetics, Faculty of Health Sciences, Igdir University, 76000 Igdır, Turkey; ^2^Research Center for Redox Applications in Foods (RCRAF), Igdir University, 76000 Igdır, Turkey; ^3^Innovative Food Technologies Development, Application, and Research Center, Igdir University, 76000 Igdır, Turkey; ^4^Advanced Pharmaceutics and Drug Delivery Laboratory, Leslie L. Dan Faculty of Pharmacy, University of Toronto, Toronto, ON, Canada M5S 3M2; ^5^Center of Experimental Medicine, Institute for Heart Research, Slovak Academy of Sciences, Bratislava, Slovakia; ^6^Molecular Hydrogen Institute, Enoch, Utah, USA; ^7^Department of Kinesiology and Outdoor Recreation, Southern Utah University, Cedar City, 84720 Utah, USA

## Abstract

COVID-19 is a widespread global pandemic with nearly 185 million confirmed cases and about four million deaths. It is caused by an infection with the severe acute respiratory syndrome coronavirus-2 (SARS-CoV-2), which primarily affects the alveolar type II pneumocytes. The infection induces pathological responses including increased inflammation, oxidative stress, and apoptosis. This situation results in impaired gas exchange, hypoxia, and other sequelae that lead to multisystem organ failure and death. As summarized in this article, many interventions and therapeutics have been proposed and investigated to combat the viral infection-induced inflammation and oxidative stress that contributes to the etiology and pathogenesis of COVID-19. However, these methods have not significantly improved treatment outcomes. This may partly be attributable to their inability at restoring redox and inflammatory homeostasis, for which molecular hydrogen (H_2_), an emerging novel medical gas, may complement. Herein, we systematically review the antioxidative, anti-inflammatory, and antiapoptotic mechanisms of H_2_. Its small molecular size and nonpolarity allow H_2_ to rapidly diffuse through cell membranes and penetrate cellular organelles. H_2_ has been demonstrated to suppress NF-*κ*B inflammatory signaling and induce the Nrf2/Keap1 antioxidant pathway, as well as to improve mitochondrial function and enhance cellular bioenergetics. Many preclinical and clinical studies have demonstrated the beneficial effects of H_2_ in varying diseases, including COVID-19. However, the exact mechanisms, primary modes of action, and its true clinical effects remain to be delineated and verified. Accordingly, additional mechanistic and clinical research into this novel medical gas to combat COVID-19 complications is warranted.

## 1. Introduction: Clinical Challenges and Dilemma of COVID-19 Treatments

COVID-19 (initially named 2019 novel coronavirus, or 2019-nCoV disease, after the first reported outbreak in 2019) has become the most widely spread global pandemic in the past century [1]. It has affected 189 countries and regions with nearly 185 million confirmed cases and about four million reported deaths worldwide as of current statistics [2]. The novel coronavirus responsible for this disease was named by the World Health Organization the Severe Acute Respiratory Syndrome Coronavirus-2 (SARS-CoV-2) for its genetic similarity to the coronavirus that caused the SARS outbreak in 2003 (SARS-CoV) [1]. While not ominously fatal as contracting SARS-CoV, COVID-19's mild symptoms and asymptomatic transmission, coupled with long incubation time and long fomite survival time of the virus have complicated epidemic control globally.

Most COVID-19 cases manifest as a respiratory illness with vague symptomatology, starting with a fever, dry cough, and fatigue, followed by shortness of breath with worsening disease. About 80% of infected people may recover from the illness without hospitalization; yet, the remainder (20%) progress to pneumonia and severe acute respiratory distress syndrome (ARDS) [3]. An estimated 5% of patients require treatment in an intensive care unit (ICU), requiring ventilation for oxygenation and intubation to support life [3]. Of these critically ill patients in ICU, approximately half eventually die of infection-associated complications, typically following multiple organ injury and failure [3]. COVID-19 complications have been correlated to underlying medical conditions, particularly older adults with hypertension, diabetes, and/or other cardiovascular diseases.

**Figure 1:**
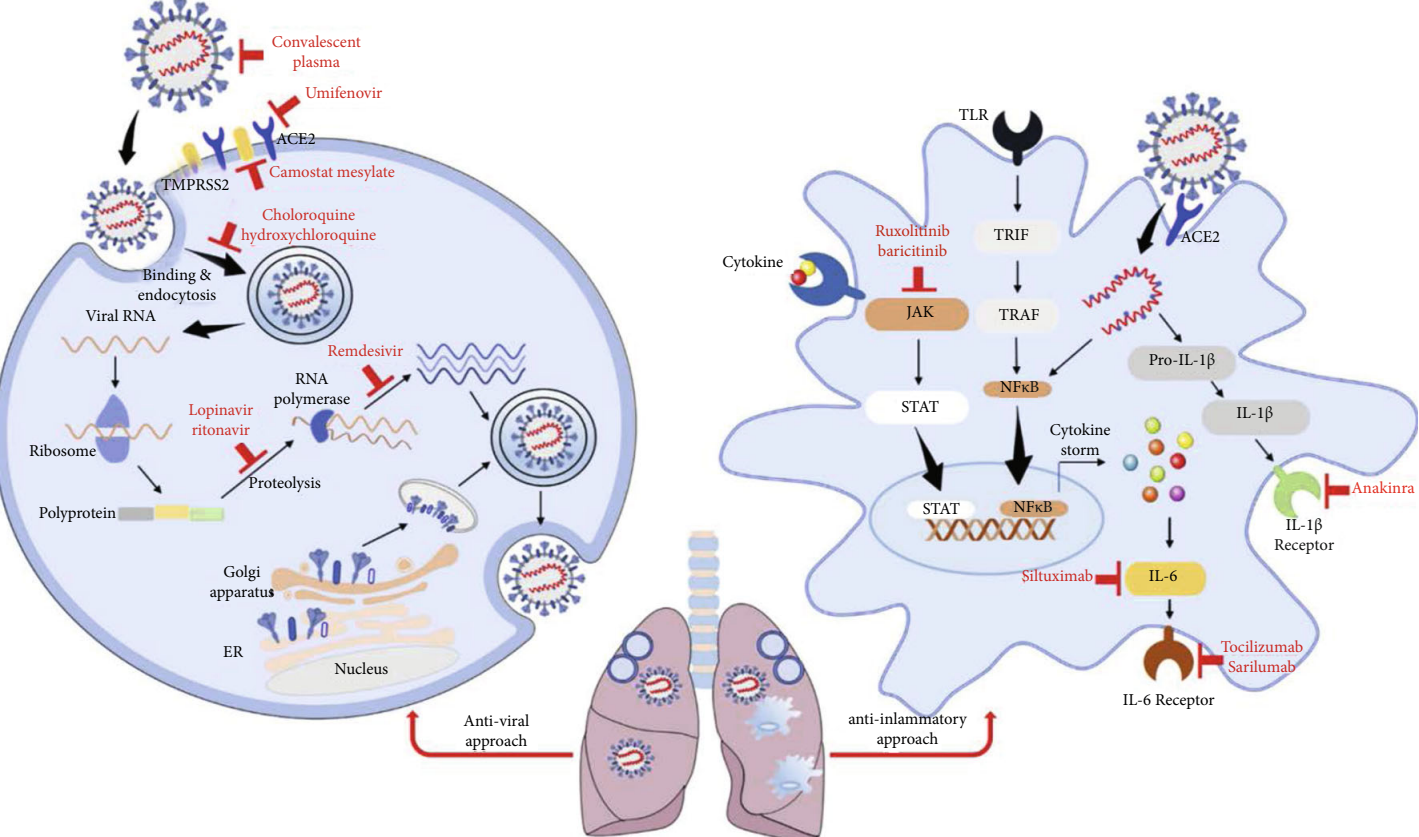
Illustration of various pharmacological therapies proposed and investigated to treat COVID-19 patients classified as two categories: antiviral approach and anti-inflammatory approach. The antiviral approach includes the use of agents that block viral binding, entry, fusion, RNA duplication, viral assembly, or exocytosis. The anti-inflammatory approach includes the application of agents that inhibit various inflammatory pathways, reduce cytokine production, and block cytokine receptors. Reproduced with permission from the publisher [4].

In contrast, cytokine storms, caused by an overactive host immune system to any infection, are most responsible for mortality in young and middle-aged patients without medical histories. Current treatment modalities, including antiviral, anti-inflammatory (Figure 1), antimalarial, immunoregulatory therapeutics, ventilation, and extracorporeal membrane oxygenation (ECMO), attempt to mitigate the sequelae caused by infection (Table 1), but they cannot fully address the upstream factors that lead to “cytokine storms,” which contribute to multiple organ failure and sudden deaths.

The coronavirus appears to exploit angiotensin-converting enzyme II (ACE2) as a receptor for cell binding and entry. ACE2 is expressed abundantly on epithelial cells in certain mucosal tissues [6]. Of note, the oral and nasal mucosa, eyes, and upper respiratory tract are the primary anatomical inoculation points for viruses that are mainly transmitted *via* aerosol droplets, propagated from human carriers in close proximity. The infection progresses to lower airways, particularly to alveolar epithelial cells that are susceptible to viral entry. When this occurs, alveolar macrophages and infiltrated immune cells are activated, which then increases oxygen consumption exacerbating alveolar hypoxia [7]. Activated alveolar macrophages also release proinflammatory cytokines within alveoli and pulmonary microvessels, which then enter the systemic circulation. Because injured lungs cannot effectively deliver oxygen or eliminate carbon dioxide from the bloodstream, systemic hypoxia (namely, hypoxemia) and hypercapnia develop. Both alveolar hypoxia and hypoxemia further induce inflammatory cascades, leading to the production of excess reactive oxygen species (ROS) and activation of hypoxia-inducible factors (HIF-1*α*), nuclear factor-kappa-light-chain-enhancer of activated B cells (NF-*κ*B), and proinflammatory cytokines [7]. Thus, oxygen inhalation and anti-inflammatory therapy are considered essential for severe COVID-19, in addition to other potentially useful therapies.

However, in severe COVID-19 pneumonia, inflammation of the respiratory tract and exudation of viscous mucus in bronchioles and alveoli make oxygenation of blood inefficient. Despite high-speed oxygen ventilation, oxygen cannot easily penetrate mucus plugs; in fact, high airflow may instead further condense the plugs. It is also speculated that the positive pressure of ventilation may break the already-fragile alveolar sacs [9]. Moreover, ventilation of highly concentrated oxygen in patients with low SpO_2_ levels may produce harmful superoxide free radicals like what happens in ischemia reperfusion.

## 2. Treatments Proposed and Investigated for COVID-19

Current guidelines for COVID-19 critical care involve general supportive measures such as hemodynamic support with a vasopressor (usually norepinephrine), corticosteroids to treat refractory shock, continuous renal replacement therapy (CRRT) or intermittent renal replacement (IRR) for acute renal failure, and mechanical ventilation to treat severe ARDS. However, the clinical benefit for patients with severe disease that requires aggressive oxygen management, such as invasive or noninvasive mechanical ventilation, high-flow oxygen, or ECMO, is uncertain. Given the high cost, procurement hurdles, and pending research, health agencies have restricted distribution to hospital systems for patients 12 years of age or older requiring supplemental oxygen without aggressive oxygen management [10].

No single pharmacotherapy has shown sufficient clinical efficacy for routine use; at the clinician's discretion, however, select patients with severe disease may receive a trial of remdesivir and/or immunomodulatory therapy (such as corticosteroids) [10].

### 2.1. Antiviral Therapies

Some preliminary studies suggest that antiretroviral remdesivir (Veklury™) may modestly shorten recovery time. However, despite its *in vitro* activity against SARS-CoV-2, its effect on mortality rate for patients with severe COVID-19 is uncertain [11–13]. Remdesivir, an adenosine analog, purportedly targets viral RNA to cause premature termination of reverse transcription [13] (Figure 1).

Other antivirals, such as lopinavir/ritonavir (Kaletra®), oseltamivir, or ribavirin, showed no clinical benefit in mortality [14–16]. Some studies combining lopinavir/ritonavir and ribavirin, however, have suggested a reduction in mortality and ARDS risk [14, 15]. Anti-infectives chloroquine and hydroxychloroquine have been studied exhaustively with clinical evidence suggesting no mortality benefit yet potential harm due to cardiac conduction abnormalities [17]. These results were negative despite their potent *in vitro* inhibitory effect on SARS-CoV-2 by raising host endosomal pH and preventing viral entry [13], though a study exploring their prophylactic role in healthcare workers is currently ongoing (NCT04334148). With similar publicity, the role of azithromycin remains contentious with the COALITION II trial, suggesting no clinical benefit when combined with hydroxychloroquine [18].

### 2.2. Immunomodulatory Therapies

Given the lack of effective antiviral treatments, some groups have investigated convalescent plasma (CP) as an interim treatment. Historically, CP has been used for various other infections (such as diphtheria, hepatitis A and B, rabies, or polio) for which at some time periods, like COVID-19, lacked any suitable pharmacological treatment [19]. In theory, immunocompetent COVID-19 survivors could produce immunoglobins as part of acquired immunity, which can then be purified and transfused. Its efficacy is heralded by reports that reinfection with COVID-19 is rare, indicating that these antibodies may be highly effective in preventing or treating severe COVID-19 [20]. While some preliminary studies have demonstrated reduced mortality, reduced oxygen requirements, and reduced viral load, with mostly minor adverse events, large-scale and high-quality clinical research is lacking [20]. Furthermore, some hypothesize that, as with infections similar to SARS and Middle East respiratory syndrome (MERS) [21, 22], conferred immunity will only last for a limited number of months and may not be effective in the long term. With the lack of viral-targeted treatments, the clinical focus has since shifted more towards preventing complications in advanced disease, with promise in treating with corticosteroids.

Corticosteroids were previously avoided due to the potential decrease in immune responses and viral clearance and increase in osteopenia and osteoporosis observed in patients with SARS and MERS [23]. Preliminary studies, however, have suggested that corticosteroids may mitigate the sequelae that lead to multisystem organ failure and lung injury observed in severe COVID-19. In particular, clinicians have closely observed the preliminary results of an open-label trial, RECOVERY (*n* = 4321), which suggested a clinically significant decrease in mortality for patients requiring oxygen and ventilation when treated with a 10-day course of dexamethasone 6 mg (NNT = 8 for ventilated patients, 34 for nonventilated oxygen therapy). No mortality benefit was observed for patients with early disease, or mild to moderate disease not requiring oxygen therapy, suggesting that dexamethasone works against the inflammatory response in later stages of disease rather than reducing the viral load [24]. Other corticosteroids were also briefly studied and are used clinically with benefit [25], but were stopped early pending the RECOVERY trial publication: these included hydrocortisone in the REMAP-CAP and CAPE COVID trials [26] and methylprednisolone [27]. Given the promiscuous anti-inflammatory nature and risks of corticosteroids, including dysglycemia, immunosuppression, latent infection reactivation particularly with *Strongyloides* [28], and agitation, research interest blossomed in pharmacotherapies that target specific anti-inflammatory pathways.

Clinicians have reported cytokine storms manifesting in patients with severe COVID-19, which has promoted additional research into molecules that target proinflammatory pathways to treat ARDS and multiorgan sequelae [29]. Some of these molecules include interleukins, such as anakinra (anti-IL-1), aviptadil (anti-IL-6 and antitumor necrosis factor (TNF)), monoclonal antibodies (anti-IL-6; tocilizumab, sarilumab, and siltuximab), and JAK inhibitors (anti-IL-6; ruxolitinib baricitinib); general anti-inflammatories such as colchicine; and steroid-sparing immunosuppressives such as sirolimus and tacrolimus. Some of these studies have suggested potential clinical benefit in COVID-19. For instance, anakinra 5 mg/kg twice daily may improve survival for patients with moderate to severe ARDS compared to a historical cohort [30]. Similarly, studies with tocilizumab for patients experiencing cytokine storms have suggested potential benefit with one or two doses of 400 to 800 mg [31, 32]. However, treatment with tocilizumab in some cases worsened COVID-19 infections, likely because of immunosuppression [33]. Similarly, studies with other molecules have suggested no clinical effect or potential harm due to immunosuppression (such as with sarilumab) [34], or insufficient power of statistical analysis to measure a mortality benefit (such as with colchicine) [35].

### 2.3. Therapies with Ancillary Benefits from Other Mechanisms of Action

Molecules targeting other host pathways are also being investigated, and many studies, as shown in Table 1, are still ongoing. Murine studies have suggested, for instance, that lung sequelae such as leukocyte infiltration and acute lung failure from the related SARS-CoV from the 2003 pandemic could be reduced with angiotensin II receptor blocker (ARB) losartan 15 mg/kg, secondarily to inhibiting ACE2 binding of viral Spike-Fc [36]. Similarly, famotidine, a histamine 2-receptor blocker used for treating acid reflux disease, may inhibit viral replication by a mechanism still being investigated. Famotidine therapy was correlated with reduced inpatient mortality or intubation [37], with some cases of reduced outpatient symptom severity reported [38]. Furthermore, recent developments in coagulopathy secondarily to cytokine storms that expose the basement membrane and activate coagulation cascades have honed research in targeting VEGF (with bevacizumab), tissue plasminogen activators (alteplase) [39], and anticoagulants (argatroban, enoxaparin, fondaparinux, heparin, and rivaroxaban) [40].

Interestingly, some molecules have been investigated based on retrospective observations of patients with polypharmacy. Many of which seem to correlate with drugs that reduce inflammation and oxidative stress. For instance, some studies have suggested that sodium-glucose cotransporter-2 (SGLT2) inhibitors, a class of multifunctional antihyperglycemics, may prevent respiratory failure associated with endothelial disruption, inflammation, and oxidative stress by purportedly reducing serum lactate production and cytokines. Studies with dapagliflozin in patients with or without diabetes are currently underway (NCT04350593) [41]. Additionally, past studies with antilipidemic “statin” drugs (e.g., atorvastatin) have suggested improved symptom management in patients with concurrent viral infections with the annual avian influenza and the 2009 H1N1. These effects may be ascribed to their anti-inflammatory, antioxidant, and ACE2-downregulatory effects, which have prompted further clinical studies with atorvastatin [42]. In fact, this projection may be supported by observations from the use of statins in, for instance, the prevention of cytokine and oxidative stress-mediated iodinated contrast-induced nephrotoxicity [43].

Some think tanks have considered incidental findings of morphine and its inhibitory effects on cytokine production, particularly in dyspneic patients. Studies have found decreased levels of IL-12, TNF, and interferons, albeit inconsistently, when morphine is used in patients with chronic obstructive pulmonary disease (COPD) [44]. Other effects observed from morphine use may be translatable to similar features in the collection of syndromes related to COVID-19. One such study explored the prevention of mitochondrial-related reperfusion injury secondarily to postmyocardial infarction percutaneous intervention. By preventing the influx of reactive oxygen species and eventual cell death, morphine could have some effect in preventing damage after restoration of oxygen status to cells [45]. Studies with the use of morphine in dyspnea have been recruiting at the time of this article (NCT04522037).

Despite the current developments outlined, and over 9100 registered clinical trials to date [5–7], the vast research vision has tunneled to individual mechanisms that include viral entry, replication inhibition, or cytokine attenuation [46].

## 3. Importance and Possible Mechanisms of Molecular Hydrogen in COVID-19 Treatment

Alveolar hypoxia, alveolar macrophages, and reactive oxygen species (ROS) cause an inflammatory response which may lead to ARDS. Excess proinflammatory cytokine secretion may further damage multiple organs. To address all of these contributing factors to cytokine storm in COVID-19, inhalation of molecular hydrogen may offer an effective solution to tackle both hypoxia and oxidative stress, thereby reducing downstream cytokine secretion. Many reports described possible mechanisms of molecular hydrogen actions against different diseases [47–60]. The majority of these reports revealed three main effects of molecular hydrogen in pathophysiology: antioxidative stress, anti-inflammatory, and antiapoptotic effects. However, these three categories also include many subgroups of different effects of molecular hydrogen observed in various studies, for example, the regulation of oxidative stress, regulation of endoplasmic reticulum stress, regulation of mitochondria, inhibition of overactivation of the immune system, prevention of apoptosis, regulation of autophagy, reduction of pyroptosis-related inflammation, protection of cells from pyroptosis, positive regulation of ferroptosis, and potential regulation of the circadian clock. In 2020, Yang et al. listed the possible mechanisms of molecular hydrogen in 10 main disease systems [48]. In 2011, Ohta summarized the diseases and the organs targeted by molecular hydrogen treatment. After the appearance of the COVID-19 disease, many global efforts were applied to fight this pandemic [61]. In China, the famous epidemiologist Dr. Zhong Nanshan has applied H_2_/O_2_ inhalation for treating more than 2000 COVID-19 patients with very positive and effective outcomes [62, 63]. Additionally, a global scientific discussion has been launched on the ResearchGate platform about the possibility of the use of molecular hydrogen in COVID-19 treatment [64]. Several articles have been published about the potential benefits of molecular hydrogen therapy for COVID-19 [48, 65, 66], including its ability to combat effects of fatigue [67]. Although its beneficial effects have been reported in the literature and demonstrated in some clinical trials, a systemic review of the properties and underlying mechanisms of molecular hydrogen is necessary to broaden the utility of its positive effects in treating COVID-19. Currently, there is no report that fully elucidates the mechanisms behind the positive influence of molecular hydrogen in COVID-19 treatment.

## 4. Physical, Chemical, and Biological Properties and Safety of Molecular Hydrogen

### 4.1. Physical Properties of Molecular Hydrogen

Hydrogen is the most abundant element in the universe especially in stars. It combines with another hydrogen atom to form molecular hydrogen, with the chemical symbol of H_2_. H_2_ is the smallest and lightest molecule with a density of 0.08988 g/L at standard temperature and pressure (STP). However, molecular hydrogen is rare in Earth's atmosphere at a level of about 0.53 ppm [68]. Hydrogen is physically characterized as a nontoxic, colorless, odorless, tasteless, and nonmetallic gas at standard temperature and pressure. H_2_ has a lower solubility in water compared to oxygen and carbon dioxide with 0.8, 1.3, and 34.0 mmol/L at 20°C, respectively [69]. The hydrogen-saturated water contains 0.78 mM (1.6 mg/L) of hydrogen at 25°C. It was estimated that 2–5% of H_2_ is lost every 3 min when hydrogen-rich water is kept in an open container [70]. To preserve the levels of hydrogen in hydrogen-rich water during storage, the product must be filled in a metal package such as aluminum as plastics are permeable to H_2_ [51].

### 4.2. Chemical Properties of Molecular Hydrogen

The earliest known chemical property of hydrogen is that it burns with oxygen to form water. Under ordinary conditions, hydrogen gas is a loose aggregation of hydrogen molecules, each molecule consisting of a pair of hydrogen atoms, to form the diatomic molecule, H_2_ [71]. Additionally, molecular hydrogen can react with many elements and compounds, but at room temperature, the reaction rates are usually so low as to be negligible due to its very high dissociation energy [72].

In food processing, H_2_ is classified as a food additive with E949, and in the European Union (EU), it is permitted in part C group I of regulation 1129/2011 additives permitted at *quantum satis* [73]. At normal temperature and pressure, H_2_ is considered a noncorrosive and not very reactive substance (inert gas). It is used to store foodstuffs in packages under modified atmosphere beside CO_2_ and N_2_, and so protects them from undesirable chemical reactions such as food spoilage and oxidation during subsequent transport and storage [74, 75]. The addition of molecular hydrogen, i.e., hydrogenation, is used to produce margarine and vegetable shortening by converting unsaturated liquid animal and vegetable oils and fats to a saturated solid form. These processes require a catalyst, and high temperatures and pressures to overcome the activation energy of the stable nonpolar covalent bond that holds the hydrogen atoms together. Moreover, hydrogen is used to reduce aldehydes, fatty acids, and esters to the corresponding alcohols.

### 4.3. Biological Properties of Molecular Hydrogen

Intestinal bacteria in humans naturally produce hydrogen at about 50 to 1,000 mg/day [76, 77] via degradation of oligosaccharides [78]. However, the amount of H_2_ produced by colonic fermentation is partially consumed by bacterial flora in the colon [70]. The ingestion of hydrogen-rich water was reported to increase both hydrogen peaks and the area under the curve (AUC) of breath hydrogen in a dose-dependent manner [79] within 10 min of ingestion [70]. It was estimated that approximately 41% of ingested H_2_ via hydrogen-rich water was kept in the body [70]. The loss of H_2_ from the skin surface is negligible (less than 0.1%). Hydrogen may be transferred to the milk when the mother drinks hydrogen-rich water [80]. H_2_ has no adverse effects on the saturation level of arterial oxygen (SpO_2_) and hemodynamic parameters [81]. The inhalation of H_2_/O_2_ mixed gas did not interfere with any vital signs of the body including respiratory rate, heart rate, blood pressure, and pulse oximetry [82].

### 4.4. Safety Property of Molecular Hydrogen

The American Conference of Governmental Industrial Hygienists classifies hydrogen as a simple asphyxiant and describes its major hazard due to its flammable and explosive properties [83]. Hydrogen is highly flammable at a range of 4-75% (*v*/*v*) in air, and it explodes in the air at the range of 18.3-59% (*v*/*v*) [84, 85]. However, the dilution of hydrogen with nitrogen lowers the risk of explosion [86]. Additionally, the autoignition temperature (the temperature at which spontaneous combustion will occur) of hydrogen is quite high, i.e., 500°C.

## 5. Redox-Related Mechanisms in the Pathophysiology of COVID-19

The cellular redox status can affect the structural composition of various sensitive components found inside or on the surface of the cell. These redox-sensitive components include many proteins/enzymes composed of sulfur-containing amino acids/peptides (SH and S-S) making them sensitive to the redox state of the environment. Methionine, cysteine (Cys), cystine, homocysteine, glutathione, and hydrogen sulfide are the common sulfur-containing compounds impacting protein regulation and cell signaling. Furthermore, the cofactors such as Fe, Zn, Mg, and Cu found in their oxidized or reduced form, make the cellular enzymes susceptible to the redox change in the environment. In the same manner, we can discuss the effect of redox value on various redox-sensitive molecules located on the surface of the cell such as enzymes, proteins, phospholipids, and saturated and unsaturated fatty acids, which could become targets for the redox change in the environment/cytoplasm. The modification in the structure of these components can directly affect different functional and structural cellular systems such as cellular transport and bioenergetics.

The cell possesses a redox homeostasis system that regulates many key functions such as protein synthesis, enzyme activity, metabolic pathways, and transport across the membrane. This redox homeostasis can be regulated by different factors such as oxidoreductases (catalase (CAT), superoxide dismutase (SODs), and glutathione peroxidase (GPXs)), metallic ions (Fe, Cu, Mg, etc.), metabolites (adenosine triphosphate/adenosine monophosphate (ATP/AMP), glyceraldehyde 3-phosphate dehydrogenase (GAPDH), and tricarboxylic acid cycle (TCA) intermediates), gaseous-signaling molecules (ROS, H_2_, H_2_S, CO, NO^•^, etc.), and internal antioxidants (ascorbate, vitamin E, *β*-carotene, urate, and thiols). Amino acids and their macromolecules, i.e., peptides and proteins, can affect and be affected by the redox state of the cytoplasm and environment. The amino acids, peptides, and proteins containing thiols (SH) form the targets for oxidants such as ROS [49]. The production of ROS and/or the change in the thiols/disulfide ratio lead to the perturbation of the intracellular redox homeostasis. This critical situation leads the cell to sense redox signaling, and thus regulate the cellular redox state [49]. When the levels of the generated ROS are high, the cell can use the redox-sensitive signaling pathways and transcription factors to upregulate genes encoding reductants such as thiols, enzymes, thioredoxin (Trxs), and glutaredoxins (Glrxs) that will reset redox homeostasis [49]. However, when the situation is more severe with very high levels of ROS, for example, during acute injury or inflammation, damage occurs to various macromolecules and cellular structures and functions, which can lead to irreversible injury and cell death. The presence of molecular hydrogen in the last case can mitigate the cytotoxic effects of ROS by reducing only the most aggressive ones, i.e., ^•^OH and ONOO^–^, without affecting the physiologically beneficial ROS-dependent signaling molecules, i.e., O_2_^•−^, H_2_O_2_, and ^•^NO, and thus, maintaining redox homeostasis of the cell [52].

The modification of the structural composition of proteins due to the change of thiol (SH) to the disulfide (S-S) form impairs molecular chaperoning, translation, metabolism, cytoskeletal structure, cell growth, and signal transduction. Additionally, the formation of disulfide bonds affects the conformation of redox-sensitive proteins [58]. It was reported that in an oxidizing medium, the sulfur group in cysteine can form intramolecular disulfide bonds creating a reversible cross-link that can be broken in the presence of a reducing agent [87]. Oxidative stress conditions are characterized by a high generation of ROS and are related to many diseases involving disulfide bond formation [87]. Thiol-disulfide reactions follow an exchangeable and rate-dependent bond rupture mechanism [87].

### 5.1. Importance of Thiols for Cellular Redox Status

Thiols have been shown to play a key role in many functional processes in cellular physiology. Glutathione (GSH), for example, was identified as a crucial intracellular antioxidant thiol that plays an essential role in protection against environmental oxidant-mediated injury in addition to its role in the redox signaling process [88]. The increase in the intracellular content of GSH leads to a decrease in the release of cytokines and chemokines from lung cells by decreasing NF-*κ*B activation. This property was related to the antioxidant activity of GSH [88]. Normally, glutathione disulfide (GSSG) represents less than 1% of the total cellular GSH pool. The perturbation to the GSH/GSSG ratio due to the excessive generation of ROS can alter signaling pathways that play key roles in many physiological responses such as cell proliferation, autophagy, apoptosis, and gene expression.

It was reported that the activation of redox-sensitive transcription factors such as nuclear factor erythroid 2-related factor 2 (Nrf2), NF-*κ*B, and activator protein 1 (AP-1) differentially regulate the genes for proinflammatory cytokines as well as the protective antioxidant genes [88]. Moreover, GSH is considered a crucial factor for the enzymatic activity of GPx, which is a major contributor to the cellular enzymatic antioxidant defense [89]. Sustained oxidative challenge leads to depletion of lung GSH along with other antioxidants forming the main reasons for many lung diseases, e.g., ARDS, chronic obstructive pulmonary disease (COPD), asthma, cystic fibrosis (CF), idiopathic pulmonary fibrosis (IPF), and neonatal lung disease [88]. Moreover, GSH levels were found to be depleted in several viral infections such as infection with HIV, influenza A virus, hepatitis C virus, and herpes simplex virus-1 [90]. On the other hand, the decrease in the levels of GSH in the lung lining fluid have been shown in various pulmonary diseases such as IPF, ARDS, CF, lung allograft patients, and patients with human immunodeficiency virus (HIV) [88]. This observation was explained by the formation of disulfide bonds due to the huge generation of ROS. Accordingly, several approaches have been studied to increase the cellular GSH levels to improve the cell's ability to cope with the increased ROS production. The administration of GSH itself has been shown to have limited therapeutic value due to its short plasma half-life, i.e., <30 min, and its inability to pass the cell membrane. Therefore, other strategies have been evaluated to increase intracellular GSH pools.

One of the most studied pro-GSH molecules is N-acetyl-L-cysteine (NAC). Roederer et al. demonstrated in 1992 that NAC inhibited HIV replication *in vitro* [91]. NAC, ascorbic acid, and vitamin E were reported to decrease both viral replication and inflammation in cells of mice infected with influenza (IV) and/or human respiratory syncytial (HRSV) respiratory viruses [92]. Although the treatment of NAC *in vitro* and *in vivo* experiments showed an increase in GSH levels that reduced the viral load by inhibiting viral replication in several viruses, e.g., influenza A (H3N2 and H5N1), the protective effect of NAC alone appeared weak or null in some models with a variation in its efficacy depending on the infecting viral strain [93]. Based on a trial study of 198 patients with COVID-19, a noticeable increase of glutathione reductase levels occurred in around 40% of COVID-19 patients [93] suggesting an increase in GSH metabolism. However, although NAC may be effective in this case, its antioxidant and therapeutic benefits may be strain specific. Therefore, clinical evidence is required before NAC supplementation can be recommended. Moreover, there is currently no COVID-specific evidence for the use of NAC [93].

### 5.2. Potential Use of Molecular Hydrogen to Improve Cellular Redox Status

A favorable GSH balance was reported to ameliorate bronchial asthma by suppressing chemokine production and eosinophil migration itself [88]. The latter authors revealed that small changes in the cellular redox status may alter signaling pathways, and the GSH/GSSG ratio can serve as a good indicator of the cellular redox state. While the increase in the GSH/GSSG leads to proliferation, the decrease in the GSH/GSSG causes apoptosis. GSH/GSSG and Cys/CySS were found to be decreased in some oxidative-related diseases such as smoking, diabetes, obesity, and pneumonia [94]. Those most susceptible to developing COVID-19 and serious illness are those with underlying pathologies such as obesity, which is associated with impaired redox and inflammatory homeostasis [95]. Another beneficial role of hydrogen in oxidative stress-related diseases may be attributed to balancing the S-S/SH in favor of thiols. Previous reports indicate that the presence of reducing agents decreased the number of disulfide bonds, resulting in a loss of cross-link-induced stability produced by the chemical microenvironment [58]. In 2012, Keten et al. reported that the stability of the disulfide bond may mildly be influenced by the redox value of the chemical microenvironment where the concentration of reducing agents can trigger various fractures in the protein by decreasing the energy barrier of disulfide rupture [87]. They performed a simulation of disulfide rupture in the presence of a hydrogen molecule, illustrating the reduction mechanism of the disulfide bond. This phenomenon was explained by the elongation of the disulfide bond leading to a weakening of the bond followed by a reduction of the sulfur atoms and fracture of the protein at the S-S bond. The authors assumed that the reaction of the hydrogen molecule with a disulfide bond occurs violently once they are near each other [87] (Table 2). However, there is no evidence that this hypothetical mechanism is responsible for the observed biological effects of molecular hydrogen at improving the GSH/GSSG ratio. However, H_2_ can increase GSH levels [96] by activating the Nrf2 pathway [97]. A nonsignificant increase in GSH, GSH/GSSG, and GSH peroxidase combined with a decrease in GSSG levels in rat livers fed with hydrogen-rich water compared to control was reported [98].

**Figure 2:**
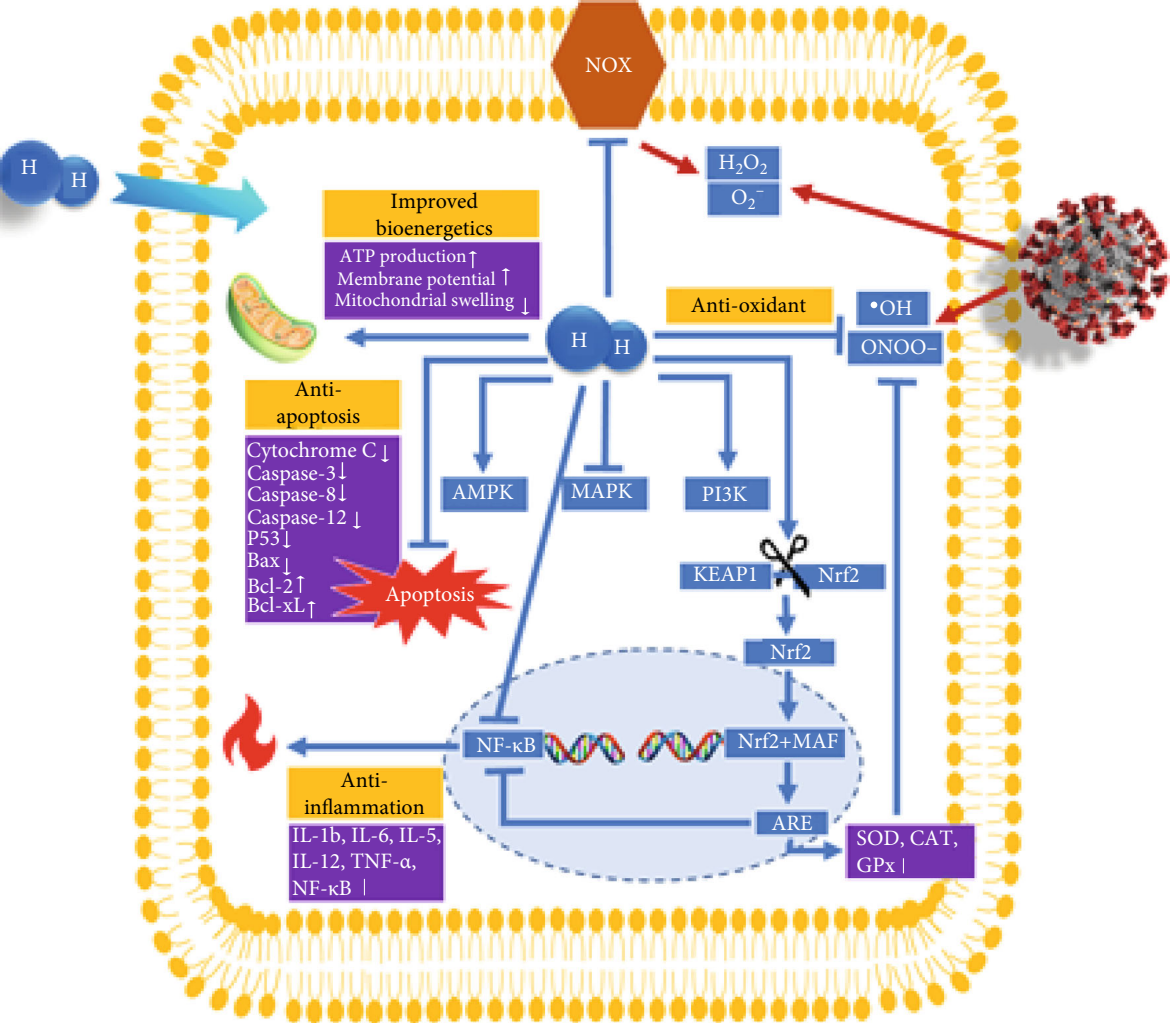
Possible mechanisms of alleviation properties of molecular hydrogen on COVID-19 patients. ^•^OH: hydroxyl radical; O_2_^−^: superoxide anion; ONOO^−^: peroxynitrite anion; H_2_O_2_: hydrogen peroxide; H_2_: molecular hydrogen; NOX: nicotinamide adenine dinucleotide phosphate (NADPH) oxidase; AMPK: 5′ adenosine monophosphate- (AMP-) activated protein kinase; MAPK: mitogen-activated protein kinase; PI3K: phosphatidylinositol 3-kinase; Keap1: Kelch-like ECH-associated protein 1; Nrf2: nuclear factor erythroid 2-related factor 2; MAF: small MAF protein; ARE: Nrf2-antioxidant response element; SOD: superoxide dismutase; CAT: catalase; GPx: glutathione peroxidase; P53: tumor protein; Bax: BCL2-associated X protein; Bcl-2: B-cell lymphoma 2 protein; Bcl-XL: B-cell lymphoma–extra-large protein; IL-12: interleukin 12; IL-1*β*: interleukin 1-beta; IL-6: interleukin 6; IL-8: interleukin 8; TNF-*α*: tumor necrosis factor *α*; NF-*κ*B: nuclear factor-kappa-light-chain-enhancer of activated B cells.

Interestingly, both endogenous and exogenous oxidants have been shown to need hours to significantly affect GSH levels in the majority of cells [88]. This is a double-edged sword because, on the one hand, the redox status stays in the range of homeostasis despite a significant amount of oxidative stress. On the other hand, by the time the GSH/GSSG ratio has changed enough to be detected, it may be too late and/or too difficult to reestablish homeostasis by pharmacological interventions. Once the GSH levels are depleted, the antioxidant redox cycling is also negatively impacted, potentially rendering pharmacological interventions or antioxidant supplementation less effective. However, premature ingestion of reducing substances either orally or intravenously may exacerbate the redox condition. In contrast, molecular hydrogen is capable of reaching any organelle in the cell within minutes and does not perturb the GSH/GSSH ratio from optimal homeostasis. Instead, H_2_ modulates signal transduction and maintains optimal redox homeostasis within the cell (Table 2). In this way, H_2_ has the ability to act as a reducing agent at low concentration with the ability to antagonize the ROS-induced deleterious effects on cell signaling [50]. H_2_ has been characterized by its ability to decrease ROS levels via upregulating superoxide dismutase (SOD) and glutathione (GSH) as well as downregulating NADPH oxidase (NOX 2) expression in a rat model [101] (Table 2 and Figure 2).

An additional but crucial role of hydrogen was found in repair processes of cell injury produced through high ROS generation. H_2_ can induce heat shock proteins (HSPs) and suppress ROS production [58]. For example, the activation of glutathione/thioredoxin systems, which reduces H_2_O_2_-induced disulfide bond formation, is another possible mechanism underlying the H_2_-induced elimination of ROS damage of inositol 1,4,5-trisphosphate receptors (IP3Rs) [58]. H_2_O_2_ is a highly reactive molecule capable of oxidizing sulfhydryl groups of cysteine and methionine in proteins and forming sulfenic acid or disulfide [49, 58]. This modification in the structure induces dysfunction of proteins leading to the impairment of many physiological processes. By this phenomenon, H_2_O_2_ was able to decrease the Ca^2+^ signal by triggering IP3R disulfide bond formation. However, the IP3R function was partially protected by treatment with H_2_ [58]. In other words, the H_2_-containing medium protected the ATP-induced Ca^2+^ signal by reducing the H_2_O_2_-induced disulfide bonds in IP3Rs.

SARS-CoV-2 infection was reported to evoke free radical-associated damage in the body by targeting different molecules. Therefore, all therapeutic means that can alleviate free radicals may be considered for COVID-19 patients to conquer the inflammation-induced burst of free radicals [104]. The rapid gaseous diffusion of H_2_ makes it highly effective for penetrating the subcellular compartments of the body. Importantly, H_2_ was identified as clinically more effective than two ROS scavengers for the treatment of cerebral infarction, i.e., edaravone and FK506, in alleviating oxidative injury [105]. In addition to the greater benefit compared to other ROS scavengers, H_2_ is considered mild enough not to affect the ROS that play essential roles in signal transduction such as H_2_O_2_, NO^•^, and O_2_^–•^ [50]. H_2_ can react with only the strongest oxidants, i.e., ^•^OH and ONOO^–^, which are considered the most reactive ROS (Figure 2). Additionally, H_2_ does not reduce the oxidized form of some biomolecules/cofactors involved in metabolic oxidoreduction reactions, e.g., NAD^+^, FAD, or the oxidized form of cytochrome C [50] (Table 2).

### 5.3. Alveolus-Related Mechanism of Molecular Hydrogen-Based COVID-19 Treatment

Pulmonary surfactants play various crucial roles in the function of alveoli. The surfactants prevent lung collapse, increase the gas exchange, and contribute to the elastic properties of the lungs. These functions of surfactants can be accomplished due to their ability to reduce the surface tension inside the alveoli. These surfactants are composed of lipids, phospholipids, and proteins synthesized and secreted by alveolar type II cells that line the alveolar surfaces of the lungs [106]. The fluid lining alveolar surfaces contains different antioxidants such as GSH, vitamin C, and ceruloplasmin, which can quench free radicals [106]. The content of GSH in the respiratory tract lining fluids (RTLFs) was reported to be subnormal in various diseases such as acute immunodeficiency syndrome (AIDS), idiopathic pulmonary fibrosis, cystic fibrosis, acute respiratory disease syndrome, and in lung allograft patients [107]. The SOD and CAT were reported to be found in both surfactant and lung epithelial lining fluid, and take part in the regulation of postnatal lung vascular development and the protection of microvasculature from ROS-induced injury [108].

The oxidative modification of surfactants due to the effect of ROS on phospholipids, lipids, proteins, and biophysical activity can lead to dysfunction and several lung diseases such as acute lung injury and acute respiratory distress syndrome [109]. ROS production can lead to an increased lipid peroxidation and destruction of the cell membrane of the alveolar epithelial cells, and an increased membrane permeability [99].

**Figure 3:**
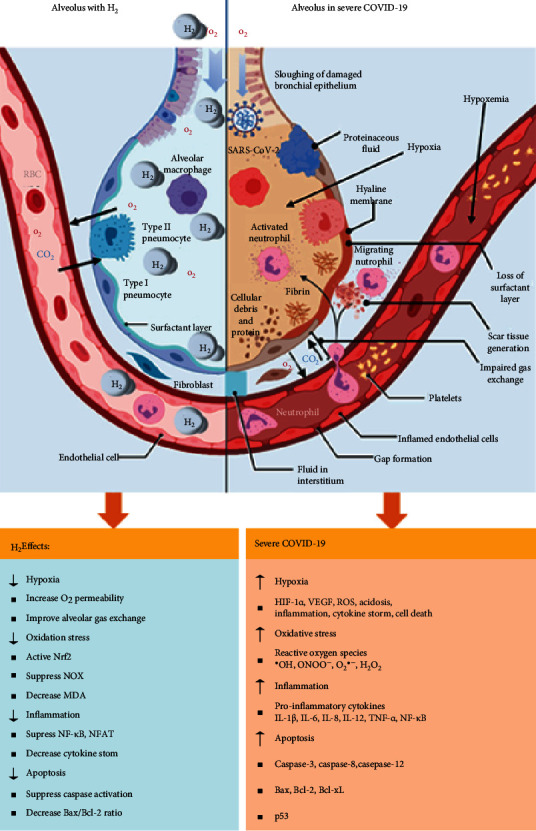
Alveolar changes due to SARS-CoV-2 infection in severe COVID-19 cytokine syndrome-induced acute respiratory distress syndrome (ARDS). Reproduced and modified from [113].

Two factors were reported to promote the oxidation of surfactant lipids. First, the excessive production of ROS makes the antioxidant defenses incapable of providing protection. Secondly, the major antioxidants in the alveoli may be excluded from the microenvironment [106]. The ROS or reactive nitrogen species (RNS), especially ONOO^−^, produced during lung injury can cause surfactant inactivation leading to increased leakage of proteins into the alveoli [110]. This latter situation prolongs the need for supplemental oxygen and assisted ventilation. It was reported that, once the SARS-CoV-2 enters the respiratory tract, it reaches the alveoli where its primary target is the type II pneumocyte, thus impairing surfactant production [111]. It was reported that both SARS-CoV-2 and SARS-CoV-1 viruses perturb alveoli to produce the major pathology in the lung, resulting in increased fluid entry, cell death, and inflammation, along with a reduction in gas exchange and levels of surfactant [112] (Figure 3).

Different antioxidants were proposed to prevent lipid peroxidation of lung surfactants such as melatonin-ebselen and vitamin E [106]. Importantly, it was reported that the continuous exposure (24 hours) to 10% hydrogen decreased the production of ROS in A549 human lung epithelial cells [80]. It was also revealed that inhalation of 2% hydrogen attenuated septic shock-induced organ injury and decreased neutrophil infiltrate in the alveoli, and reduced alveolar damage [99]. On the other hand, inhalation of H_2_/O_2_ mixed gas has been shown to reduce the inspiratory effort in patients with acute severe tracheal stenosis [82]. Moreover, hydrogen-rich water was reported to protect against the alveolar destruction attenuating the oxidative DNA damage and swimming-induced pulmonary edema (SIPS) in the lungs of COPD model mice [102]. Furthermore, hydrogen-rich water was found to attenuate lung injury by inhibiting lipid peroxidation [103]. Hydrogen-rich saline was also reported to reduce ROS production in alveolar epithelial cells, attenuate the alveolar epithelial barrier damage, improve alveolar gas exchange, and reduce cell damage caused by alveolar epithelial cell apoptosis and excessive autophagy [60] (Table 2).

## 6. Conclusion and Perspectives

An explanation for the advantageous effects of molecular hydrogen in COVID-19 treatment is related to the different properties of molecular hydrogen: (1) the small molecular size and nonpolarity of H_2_ allow it to rapidly permeate the tissues and cells, (2) it can selectively reduce only the cytotoxic ROS, (3) it can suppress the excessive production of otherwise good ROS, (4) it can suppress proinflammatory cytokines, (5) it can induce cytoprotective heat shock proteins, (6) it can improve mitochondrial bioenergetics, and (7) it has no known toxic effects even at very high levels [114]. These properties may explain the improvement in the conditions of COVID-19 patients treated by inhalation of H_2_/O_2_ mixed gas (67% H_2_/33% O_2_), who felt reduction in chest pain and cough, and easier deeper breathing and comfort sensation [62, 63]. The positive results of the pilot study led Dr. Zhong Nanshan, the epidemiologist who discovered the SARS virus (SARS-CoV-1) in 2003, to recommend the H_2_/O_2_ inhalation therapy for COVID-19 patients [115] and prompted more clinical trials using H_2_/O_2_ mixed gas [116–118].

Currently, there are twenty registered clinical trials on the use of H_2_ for COVID-19. Of these, four are registered at the Centre for Evidence-Based Medicine (Oxford) using H_2_/O_2_ mixed gas inhalation [116], five clinical trials are registered at ClinicalTrials.gov of the US National Library of Medicine for inhalation [118], eight clinical trials are registered at ICTRP (WHO) with six for inhalation and two trials for hydrogen-rich water [8], and three clinical trials, related to the use of either inhalation or ingestion of hydrogen-rich water, are registered at the Chinese Clinical Trial Registry center [117]. Up to date, the reported benefits of H_2_ therapy in COVID-19 patients are limited to the symptomatic description. To expand the utility of H_2_ therapy in COVID-19, more thorough understanding of the underlying mechanism of H_2_ in patients is required. Therefore, accurate analysis of a broad spectrum of biomarkers is highly recommended to delineate the correlation between clinical and biochemical presentations and the proposed biological effect of H_2_.

According to the report of WHO, data from China and around the world suggest that the majority of people with COVID-19 have a mild illness, about 15% of them have a severe illness requiring oxygen therapy, and 5% are critically ill requiring mechanical ventilation. Owing to the widespread transmissibility and emergence of more infectious variants of SARS-CoV-2, many hospitals have been overwhelmed by the crush of new COVID-19 patients and have exhausted ICU beds and ventilators in some regions. Therefore, an alternative yet effective treatment, e.g., H_2_/O_2_ gas inhalation, would ease the pressure on hospitals and prevent severe illness of COVID-19 patients.

The medical model of H_2_/O_2_ mixed gas machine is small, portable, and safe [119]. It costs about one-tenth of the price of a ventilator. The H_2_/O_2_ inhalation treatment may be performed in regular wards or by outpatients at home isolation using a portable H_2_/O_2_ generating and inhalation device. This kind of treatment may reduce hospitalization time for a high number of patients. This strategy could decrease the pressure of massive patient numbers on hospitals. It is important to mention that, although molecular hydrogen is not considered a drug, its intake in different ways such as drinking hydrogen-rich water or inhaling H_2_/O_2_ gas may be beneficial in preventive medical health in addition to its therapeutic use. Due to the high safety profile and favorable preliminary results in preclinical and clinical studies, application and additional research of molecular hydrogen therapy for COVID-19 are encouraged.

## Figures and Tables

**Table 1:** Selected treatments investigated in clinical trials for COVID-19.^∗^.

Antiviral	Anti-inflammatory	Anticoagulation and antivasculopathy
Arbidol	Acalabrutinib	Argatroban
Azithromycin (antibiotic)	Anakinra	Alteplase
Camostat mesilate (reduce viral infection)	Aviptadil	Alteplase
Chloroquine or hydroxychloroquine	Baricitinib (janus kinase (JAK) inhibitor)	Acetylsalicylic acid
Clevudine	Chlorpromazine	Atorvastatin (HMG-CoA inhibitor)
Darunavir/Cobicistat (Prezcobix; Rezolsta)	Colchicine	Bevacizumab (antivascular endothelial growth factors (VEGF))
Favipiravir (Avigan®)	Deferoxamine	Clopidogrel
Interferon	Dexamethasone	Crizanlizumab (vasculopathy)
Ivermectin plus nitazoxanide	Dornase alfa (Pulmozyme®)	Dapagliflozin (sodium-glucose transporter-2 inhibitor)
Lactoferrin	Duvelisib	Enoxaparin
Lopinavir-ritonavir (Kaletra®)	Eculizumab	Fondaparinux
Nafamostat (blocks TMPRSS2 activation and SARS-CoV-2 cell entry)	Famotidine	Heparin
Oseltamivir	Hydrocortisone	Losartan (angiotensin II receptor blocker (ARB))
Remdesivir	Imatinib	Nitric oxide (inhalation) Nicotine
Umifenovir	Infliximab	Ramipril (angiotensin-converting enzyme inhibitor (ACEi))
	Isotretinoin	Rivaroxaban (direct oral anticoagulant (DOAC))
	Leflunomide	Sulodexide
	Methylprednisolone Morphine	Telmisartan (ARB)
	Ozanimod	Valsartan (ARB)
	Plitidepsin	
	Prednisolone	
	Ruxolitinib (JAK inhibitor)	
	Sarilumab	
	Sirolimus	
	Tocilizumab (IL-6 inhibitor)	
	Tofacitinib (JAK inhibitor)	
*Antioxidant treatment*	*Traditional Chinese medicine*	*Oxygen therapy*
Vitamin A	Single herbs	Oxygen inhalation
Vitamin C	Chinese patent formulas	Mechanical ventilation
Vitamin D	Chinese herbal compounds	Prone position ventilation
Vitamin E		Hyperbaric oxygen therapy
Glutathione		Oxyhydrogen inhalation *via* a nebulizer
N-Acetyl-L-cysteine (NAC)		
Melatonin		
Zinc		
*Vaccine and antibodies*	*Extracorporeal membrane oxygenation support*	
mRNA, recombinant protein, vector	Oxygenation, removal of CO_2_, filtrating proinflammatory cytokines via a filter	
Pamrevlumab		
Anti-SARS-CoV-2 convalescent plasma		

^∗^Selected from 9149 total COVID-19 studies, including 6115 from COVID-19 NIH registered clinical trials [5] and the rest registered outside of the USA found from WHO International Clinical Trials Registry Platform (ICTRP) database [6–8].

**Table 2:** Summary of some possible mechanisms related to the positive effects of molecular hydrogen in different diseases and COVID-19 treatment.

Possible Mechanism	Type of Study	Principle	Reference
Molecular properties-related mechanisms	In vivo	Unlike most antioxidants, can penetrate biomembranes and diffuse into the cytosol, mitochondria and nucleus and reach cell organelles	[50]
		Has a rapid gaseous diffusion rate making it highly effective for reducing cytotoxic radicals	
Redox-related mechanisms		Regulates the redox homeostasis after a ROS-related dissipation stage	
		Mild enough not to disrupt metabolic oxidoreduction reactions or interrupt ROS-induced disruption of cell signaling	
		Selectively reduce the strongest cytotoxic oxidants, ^•^OH and ONOO^–^; whereas, the biological useful oxidants such as superoxide, hydrogen peroxide, nitric oxide are not altered	
		Protects nuclear DNA and mitochondria	
		Protects cells and tissues against strong oxidative stress	
		Decreases production of ROS	
	in silico	Reduces the reversible cross-linked intramolecular disulfide bonds formed after an oxidative stress e.g. ROS	[86]
		Decreases the energy barrier of disulfide rupture	
	In vivo	Balances the S-S/SH in favor of thiols	[58]
		Protects Inositol 1, 4, 5-trisphosphate receptors (IP3Rs) function	
		Protects the ATP-induced Ca^2+^ signal by reducing the H_2_O_2_-induced disulfide bonds in IP3Rs and restores protein function	
		Activates glutathione/thioredoxin systems involved in the modulation of disulfide bond formation during oxidative stress leading to reduced H_2_O_2_-induced disulfide bond formation	
		Repairs the processes of cell injury produced through high ROS generation	
	Animal	Mitigates the oxidative damage	[98]
		selectively reduces ^•^OH attenuating ischemia/reperfusion-Induced organ damage	
		Increases superoxide dismutase (SOD) activity against ROS-mediated cellular damage	
		Increases activities of antioxidant enzymes	
		Can significantly decrease levels of oxidative products	
	Human	Induces superoxide dismutases (SODs) activity to quench ROS production	[99]
	Human	Decreases ROS levels via upregulating superoxide dismutase (SOD) and glutathione (GSH) as well as downregulating NADPH oxidase (NOX 2) expression	[100]
	Animal	Decreases oxidative damage	[98]

Inflammatory reactions and apoptosis-related mechanisms	Animal	Inhibits the over-expression of inflammatory factors (IL-6, IL-8 and TNF-*α*)	[98]
		Downregulates the expression of proapoptotic Fas proteins	
		Up-regulates the expression of the anti-apoptotic protein Bcl2	
		Ameliorates LPS-induced bronchopulmonary dysplasia	
		Reduces LPS-induced oxidative stress production	

Lung and alveoli-related mechanisms	Animal	Ameliorates LPS-induced suppression of genes encoding fibroblast growth factor receptor 4 (FGFR4), VEGFR2, and HO-1, as well as LPS-induced overexpression of inflammatory marker proteins (TNF*α* and IL-6)	[79]
		Suppresses the induced expressions of inflammatory marker proteins (TNF*α* and IL-6)	
		Reduces ROS production in alveolar epithelial cells	
	Animal	Attenuates septic shock-induced organ injury	[98]
		Decreases neutrophil infiltrate in the alveoli	
		Reduces alveolar damage	
		Reduces levels of high-mobility group box 1 in serum and lung tissue improving the survival rate of mice with sepsis	
		Reduces the levels of IL-6, IL-8 and TNF-*α*	
		Down-regulates the levels of Fas protein and up-regulates the levels of Bcl2 protein, which may inhibit ALI by inducing apoptosis, and may protect lung function	
		Effectively prevents enterogenous sepsis	
		Significantly decreases the level of MDA and MPO	
	Animal	Protects against the alveolar destruction attenuating oxidative DNA damage and SIPS in the lungs	[101]
		Decreases the markers of oxidative DNA damage such as phosphorylated histone H2AX and 8-hydroxydeoxyguanosine, and senescence markers such as cyclin-dependent kinase inhibitor 2A, cyclin-dependent kinase inhibitor 1, and b-galactosidase	
		Restores static lung compliance	
		Reduces airspace enlargement and parenchymal destruction	
		Attenuates cigarette smoke-induced oxidative DNA damage and premature senescence in the lungs	
	Animal	Enhances phagocytic activity of alveolar macrophages	[102]
		Attenuates lung injury	
	Animal	Attenuates alveolar epithelial barrier damage	[60]
		Improves alveolar gas exchange	
		Reduces cell damage caused by alveolar epithelial cell apoptosis and excessive autophagy	
	Human	H_2_/O_2_ mixture relieves dyspnea and alleviates patient discomfort during the perioperative period	[81]

Small intestine injury-related mechanisms	Animal	Protects the intestinal mucosa from mechanical injury	[98]
		Reduces the pathological changes of the small intestine	
		Inhibits bacterial translocation	
		Protects the function of other organs in the body	
